# Immune Cell Metabolic Fitness for Life

**DOI:** 10.3390/antib11020032

**Published:** 2022-04-30

**Authors:** Kevin S. Bittman

**Affiliations:** Agilent Technologies, Inc., Santa Clara, CA 95051, USA; kevin.bittman@agilent.com

**Keywords:** CAR T, immunotherapy, bioenergetics, metabolism

## Abstract

Adoptive cell therapy holds great promise for treating a myriad of diseases, especially cancer. Within the last decade, immunotherapy has provided a significant leap in the successful treatment of leukemia. The research conducted throughout this period to understand the interrelationships between cancer cells and infiltrating immune cells winds up having one very common feature, bioenergetics. Cancer cells and immune cells both need ATP to perform their individual functions and cancer cells have adopted means to limit immune cell activity via changes in immune cell bioenergetics that redirect immune cell behavior to encourage tumor growth. Current leading strategies for cancer treatment super-charge an individual’s own immune cells against cancer. Successful Chimeric Antigen Receptor T Cells (CAR T) target pathways that ultimately influence bioenergetics. In the last decade, scientists identified that mitochondria play a crucial role in T cell physiology. When modifying T cells to create chimeras, a unique mitochondrial fitness emerges that establishes stemness and persistence. This review highlights many of the key findings leading to this generation’s CAR T treatments and the work currently being done to advance immunotherapy, to empower not just T cells but other immune cells as well against a variety of cancers.

## 1. In the Beginning

The idea of using the body’s own immune system to fight cancer has a long history, far pre-dating the discovery of many of cells identified as representing the immune system [1]. Among the earliest observations relating the immune system to tumor regression came in the 1860’s from two German physicians working independently, Fehleisen and Busch, who noted tumor regression in patients with erysipelas, a skin disorder caused by the bacteria Streptococcus pyogenes [1]. In the 1890’s, a young surgeon, William Coley, made similar observations as Fehleisen and Busch in ill patients. It seemed that a bacterial infection helped reduce the size of a tumor. Coley went so far as to infect cancer patients with Streptococcus pyogenes with amazingly positive results at a time when little was known about the immune system [1,2]. Unfortunately, this lack of understanding nudged scientists away from using infection as a tool to treat cancer. It took nearly 100 years, the discovery of signaling molecules like interferon, the identification and classification of cells of the innate and acquired immune system, and a better understanding of antibodies before immunotherapy could really emerge as an accepted option [1].

**Figure 1:**
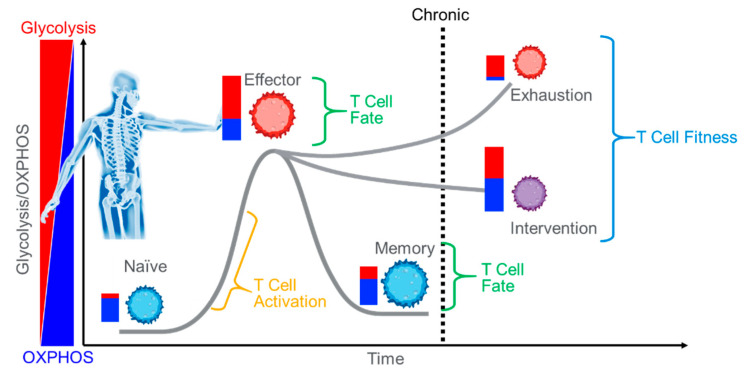
Immunotherapy. Using an individual’s own immune system to fight disease must overcome numerous obstacles such as anergy and exhaustion. Intervention methods often target metabolism. Improving T Cell capacity and efficiency of ATP production is both an approach and a validation target.

What Coley and his contemporaries lacked was an understanding of the mechanisms of action that led to cancer suppression. Infection with bacteria to treat cancer at that time was considered a dangerous, unpredictable wild shot. But, through the 1970s, 1980s, and 1990s, a more complete understanding of immune cell function and signaling developed. Coupled with a greater understanding of genetics, immunotherapy becomes a viable, powerful option for treating cancer. This approach is no longer about merely stimulating the immune system to fight cancer, it is now about modifying the immune cells in the dish, and then using them to better fight cancer, i.e., Adoptive Cell Transfer [3,4]. Tumor Infiltrating Lymphocytes (TILs), engineered T Cell Receptor therapy (TCR), and Chimeric Antigen Receptor T Cell Therapy (CAR T) are the modern forms of Coley’s immunotherapy. These treatments modify an individual’s own immune cells in order to fight cancer (Figure 1). At the heart of this rebirth lies a rather dynamic immune cell, the lymphocyte.

It’s been known for some time that cancer cells express unique epitopes that can be recognized by immune cells, thus making the immune system an attractive tool for destroying cancer [4,5]. Monoclonal antibodies, radio immunotherapy and Bacille Calmette-Guérin (BCG) have all had some success in modern day treatment of various cancers [1,6,7,8]. The issues faced by these approaches include tumor immune avoidance, weak immunogenicity, and poor persistence of the treatment. Because the acquired immune cells are highly specific and retain memory, they are at the top of the list as key soldiers to fight cancer. Now the question is, what to modify to improve the outcomes? How do you overcome weak immunogenicity or poor persistence? The answer may rely on bioenergetics.

## 2. Basic of Bioenergetics

Bioenergetics is how living cells manage energy levels to perform various functions, most notably catalyzing enzyme reactions. The primary energy currency is adenosine triphosphate (ATP), and phosphorylation is the process. Cells only keep what they need, ATP is not stored, so regulation of ATP production is a critical process to promote healthy cellular function. This is especially true among dynamic cell populations, such as immune cells that will change behavior in response to an invading pathogen. Activation leads to expansion, cytokine production and signaling, migration, differentiation, antibody production, apoptosis, etc. These activities require ATP and require ATP quickly, lest the pathogen overwhelm the host organism before a defense is mounted. Understanding the pathways, pathway choices, and flux of substrates that fuel energy production is central to understanding immune cell function, especially in a competitive environment. This knowledge will fuel ideas for future treatments.

There are two main energy producing pathways, each with unique characteristics. Glycolysis, occurring in the cytosol, produces two molecules of ATP per molecule of glucose through a series of enzymatic reactions. The end product of glycolysis is two molecules of pyruvate. The second main energy producing pathway is the mitochondrion, a highly specialized organelle with its own source of DNA. Within the mitochondria is the tricarboxylic acid cycle or Krebs cycle, another series of enzymatic reactions that runs in a loop with numerous intermediaries and products including reducing equivalents, GTP, amino acids, and carbon dioxide. The reducing equivalents, NADH and FADH_2_, produced by the TCA, feed into the respiratory chain, five linked super complexes that oxidize substrate to form from 30–38 molecules of ATP from one molecule of glucose. There are numerous substrates that the mitochondria can oxidize to produce these reducing equivalents, with palmitate, glucose/pyruvate, and glutamine being the most common, and the different substrates produce different amounts of ATP. This provides flexibility. The respiratory chain requires oxygen to produce ATP. Glycolysis, on the other hand, does not require oxygen. At any given moment, a cell usually has access to either of these pathways to manage the ATP pool. The choices cells make to manage ATP production, redox balance, and reactive oxygen species can be surprising, are influenced by cell-to-cell signaling, environmental conditions, cell health, and can dictate the functioning of the cell. Bioenergetic pathways offer multiple solutions to problems cells may encounter, such as tumor emergence, and as such also represent systems that may be manipulated to improve disease outcomes.

## 3. The Emergence of T Cell Fitness—The Opposite of Exhaustion

**Figure 2:**
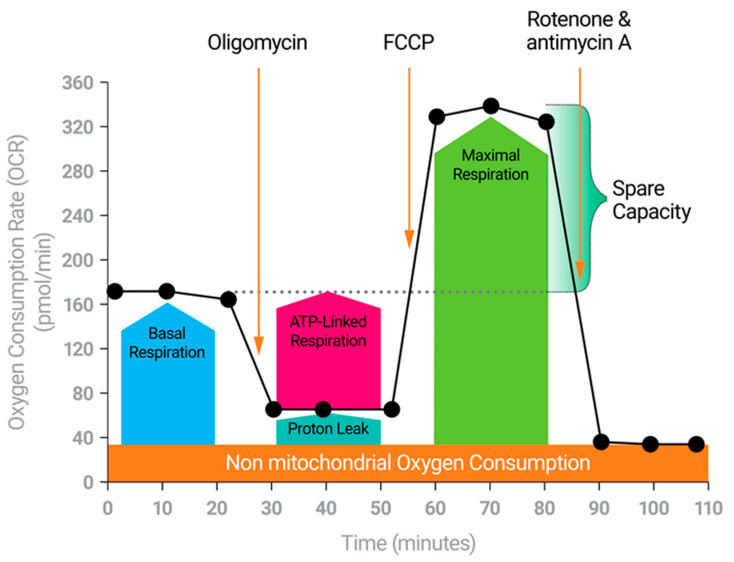
The Cell Mito Stress Test. The Cell Mito Stress Test measures the key parameters of mitochondrial function including basal respiration, ATP-linked respiration, maximal respiration, Spare Respiratory Capacity, and non-mitochondrial respiration.

So why is bioenergetics important to CAR T manufacture? It is largely accepted that dynamic cells, cells that drastically change behavior following environmental stimulation, such as lymphocytes, will require increased ATP to drive the reactions that provide new functionality [9]. How cells meet the energy demand, the nature of this demand, and whether this is a cause or an effect is under intense scrutiny, and the answers will provide the insights necessary for future cancer therapies. As far back as 1973, Roos and Loos demonstrated increased glycolytic and mitochondrial ATP production in antigen stimulated lymphocytes [10,11]. In fact, they estimated that following activation, 85% of the ATP produced by a lymphocyte came from the mitochondria [10]. The catabolic response to antigen stimulation occurs within seconds, highlighting the importance of ATP for activities such as proliferation and expansion [9]. Glucose uptake, increased glycolytic activity, and increased respiration are among the first effects of T cell receptor stimulation [9,12,13,14,15]. Further, the various T Cell subtypes differ in the nature of energy maintenance and production and offer some key findings to drive future breakthroughs in immuno-oncology. Naïve CD8+ T Cells, for example, are relatively quiescent catabolically when compared with effector-stimulated CD8+ T cells, where glycolysis increases. Following contraction, some CD8+ T cells remain in circulation with antigen specific memory. These cells appear metabolically quiescent, too. However, CD8+ memory T cells exhibit a unique mitochondrial phenotype uncovered with the Agilent Seahorse Cell Mito Stress Test (Figure 2). They possess increased Spare Respiratory Capacity (SRC) when compared with their counterparts [14,15]. Results are similar with CD4+ T cells [13]. The SRC is an indication of the potential capacity of the cells to produce mitochondrial ATP. It represents what the cell could produce if it were needed [16,17], and coincides with stemness and persistence and is often referred to as Immune Cell Fitness [15,18,19,20,21]. Taken together, these data have spurred intense research efforts to identify the factors that promote T cell activation, stemness, expansion, persistence, and memory in the tumor microenvironment. Mitochondrial fitness is a requirement and makes for an excellent marker. So, although the original concepts for engineering a CAR T cell for fighting cancer focused on the intracellular signaling that induced activation and persistence [5,22], without the right bioenergetics, the newly made CAR T cell will not get the job done. In fact, the cancer recognizes this. SRC is a target for cancer cells to drain from infiltrating T cells [23,24], possibly one of the root causes of T cell exhaustion [25]. So, robust functional mitochondria are a must for the CAR T cell.

## 4. The Hostile Tumor Microenvironment

Within the tumor micro environment, a number of tumor-initiated processes such as extracellular vesicular signaling, tumor nanotubule mediated signaling, and other tumor secreted factors can induce changes in immune cell behavior to favor tumor growth [23,26,27]. Exhaustion, originally characterized by Zajac et al. [28] in response to epitope specific chronic viral infection of CD8+ T cells, represents the progressive loss of effector function. Immune cell exhaustion is a common result from the steady growth of many tumors [29,30,31,32]. Microarray analysis has identified 490 genes differentially regulated in exhausted CD8+ T cells when compared with naïve CD8+ T Cells. These differences include increased gene expression for differentiation and inhibitory genes (e.g., PD-1), reduced expression of signaling related genes, and major changes in metabolic-related genes, including reduced expression of TCA cycle intermediates [33]. Metabolically, it has been demonstrated that chronic CD8+ T cell activation leads to increased reliance on glycolysis and decreased utilization of mitochondria for ATP production. In fact, the deficit appears to be related to impaired TCA cycling, consistent with Wherry et al.’s findings [25,32,33]. Further, chronically stimulated CD8+ T cells exhibit much lower rates of respiration, less coupled respiration, and less spare capacity when compared with acutely stimulated CD8+ T cells. ATP/AMP ratios decrease, and mitochondrial complex III derived Reactive Oxygen Species (ROS) levels increase. ROS then contribute to exhaustion through activation of the nuclear factor of activated T cells (NFAT), a transcription factor involved in T cell exhaustion [25,34]. Treatment of chronically activated cells with the anti-oxidant N-acetylcystein (N-AC) reverses the effects of exhaustion and promotes a healthy bioenergetic phenotype, i.e., increased coupled respiration and spare respiratory capacity. Thus, mitochondria appear to play a more direct role in exhaustion than previously thought.

Does exhaustion occur in engineered T cells? The genetic engineering process is designed to overcome this shortcoming, but despite efforts to design highly anti-cancer CAR T cells, not all patients respond equally. Further, solid tumors pose unique challenges that CAR T therapy has yet to overcome. Exhaustion is one possible reason for poor T Cell responses [35]; however, there are many factors to consider. The tumor microenvironment is unfavorable to the metabolic needs of invading immune cells. Low oxygen, high acidity, and limited resources blunt the potential for invading lymphocytes [36,37,38]. The pH of the environment is a critical factor, and when acidic, damages healthy tissue, dampens immune responses, and primes the area for metastasis and invasion [39,40,41,42]. This may be a key reason why CAR T cell strategies struggle with solid tumors. Tumors can produce acid from several sources, most notably the extrusion of lactate following glycolysis [39], and both acidity and lactate have been shown to reduce T cell activity [38,42,43]. Interestingly, as effector T cells are highly glycolytic, they may suppress themselves.

Approaches addressing glycolysis and lactate should be considered in CAR T therapy. Simple neutralization of the tumor microenvironment, for example, has been demonstrated to slow prostate tumor growth [40], an approach that also appears to restore T cell function in a lactate-rich environment [43]. Further, limiting glycolytic activity among tumors increases CD8+ T cell infiltration during α-CTLA-4 treatment while destabilizing T regulatory cells [44]. Work on lactate and pH in the tumor microenvironment is far from complete. While there are numerous approaches to managing lactate levels, such as inhibition of lactic acid dehydrogenase, inhibition of lactate transport, and neutralizing environmental pH, lactate is a critical metabolite whose role in immune cell and cancer cell bioenergetics is complex [38]. Recently, Ye et al. have employed a gain of function screen in primary T cells using CRISPR to express proteins not normally present in primary T cells and then categorized the hits by effector function. They identified the gene proline dehydrogenase 2 (*PRODH2*) as a potential new target to help drive CAR T cell efficacy, an enzyme that produces the redox coenzyme FADH_2_. Driving expression of *PRODH2* in CD22 CAR T leads to enhanced cancer killing, and increased lifespan in mouse leukemia models. Interestingly, lactate production increases in the PRODH2 CAR T cells [45]. Exploration of this lactate conundrum might help advance CAR T efficacy against solid tumors.

## 5. Mitochondria Wanted—By T Cells and Cancer Cells

Mitochondria play a critical, and sometimes unexpected, role in the tumor microenvironment. Take neutrophils, for example. These early responders possess few mitochondria, however, when they engage cancer, mitochondrial expression occurs, and the cells revert to an immature-like status. With an abundance of mitochondria, and under the influence of the tumor microenvironment, the immature neutrophils produce high levels of Reactive Oxygen Species (ROS) that appear to inhibit T cell function while promoting tumor growth [46]. Another example involves the hijacking of mitochondria from invading T cells by tumor cells. Saha et al. [23] demonstrate that MDA-MB231 cells extend nanotubules to link with CD3+/CD8+ T cells and suck the mitochondria from the T cells. The Seahorse data indicate the changes in function for each cell. Mitochondrial SRC decreases in the infiltrating T cells and increases in the breast cancer cells, and the T cells begin to die off [23]. Both of these scenarios, neutrophils and T Cells, involve cancer cell manipulation of immune cell mitochondria. In the case of T cells, this has a profound effect on SRC. In ovarian cancer, the ascites fluid perfusing the tumor has been shown to decrease the SRC of infiltrating lymphocytes. In this scenario, unidentified components within the ascites reduced both glucose uptake and glutamine uptake. This leads to endoplasmic reticular (ER) stress, presumably via a lack of *N*-linked protein glycosylation. Preventing ER stress restores glutamine uptake among tumor infiltrating lymphocytes, restores SRC, promotes T cell survival, and enhances anti-tumor immune activity [47]. What should be noted here is that SRC is not the result of a single pathway or single event. It is a very sensitive measure of T cell fitness that is responsive to the available oxidizable substrates as well as the abundance of mitochondria present in the T cell. So, what is the relevance of mitochondria for T cell function? Mitochondria may be at the crux of it, and this is why technologies such as Chimeric Antigen Receptor T Cells (CAR T) rely heavily on not just mitochondria, but mitochondrial fitness, or SRC.

**Figure 3:**
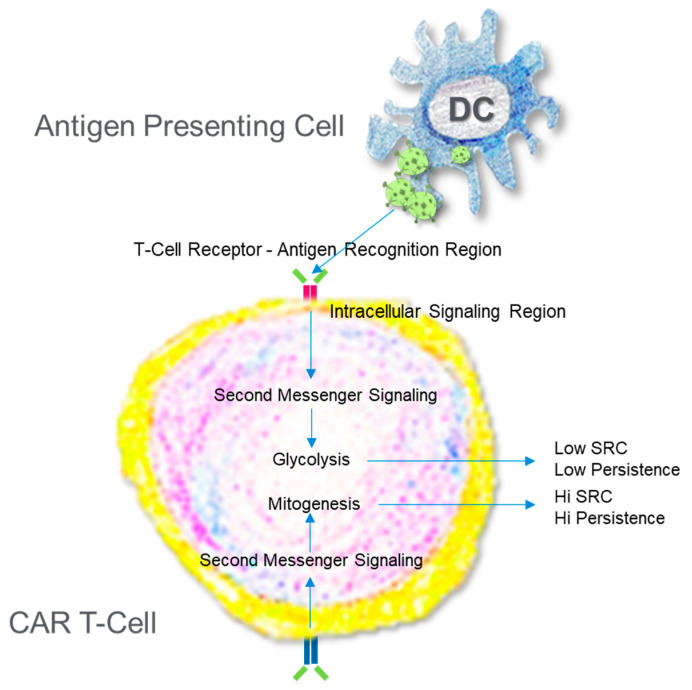
Engineering a Chimeric Antigen Receptor T Cell. T Cells are collected from individuals and then reprogrammed via gene editing to improve the sensitivity to antigen and promote the production of ATP. The path chosen to increase ATP production beyond what a normal activated T cell can do dictates the persistence.

A successful CAR T cell engineering strategy seeks to produce a T cell with high recognition for a tumor antigen, robust activation, and significant persistence, thus providing tunability, fidelity, and longevity [4,22]. These latter two conditions, activation and persistence, are highly dependent on bioenergetics (Figure 3). Like the CD4+ and CD8+ memory T cells, effective CAR T cells exhibit significant mitochondrial SRC, and this is not a fleeting observance. Weeks after construction, high SRC remains [19]. Even after 10 years, persisting CAR T cells showed enrichment in several mitochondrial-associated genes [48]. SRC is a driver for evaluating and predicting the value of a CAR T construct and the technique to produce it. Alizadeh et al. [49] examined the role of various cytokines for the expansion of CAR T cells. Interleukin-2 is a favorite for expansion of the CAR T cell population, however, repeated exposure to IL-2 can result in T cell differentiation and impaired activity [50,51]. There are several cytokines to examine in addition to IL-2, and cytokine exposure often leads to metabolic changes, so functional mitochondrial assessment can provide illuminating data regarding the mitochondrial health and anti-tumor potential of the CAR T cell. Expansion with IL-15 produces CAR T cells, demonstrating high SRC (metabolic fitness), stemness, persistence, and superior anti-tumor activity when compared to cells expanded with IL-2 [49]. Another approach involves altering the signaling cascades or design a novel “superkine”. Mo and colleagues [20] modified the signaling induced via IL-2 in CD8+ T cells by producing a partial IL-2 agonist, H9T, with disproportionate affinity for the IL-2 receptors IL-2Rβ and IL-2Rγ, thereby modulating intracellular signaling to promote T cell stemness. Expanding the CAR T cell population with the partial agonist led to greater antitumor activity than with IL-2 alone. These CAR T cells also exhibited higher SRC than their counterparts. Further, restricting glycolysis promoted stemness, thus further solidifying a relationship between bioenergetics and CAR T cell effectiveness [20]. Gautam et al. [18] take another approach, looking directly at transcription factors that can regulate cell fate, in this case c-Myb, a proto-oncogene involved in cell fate determination. When deleted, pmel-1 CD8+ T cells differentiate and contract much sooner than wildtype cells. These cells display the hallmarks of effector cells, including reduced SRC [18]. This metric, SRC, has proven to be helpful in providing researchers a mechanism to assess their methodology for producing the best possible CAR T cell. Why is SRC important? Where does it come from?

## 6. Spare Respiratory Capacity—A Measure of T Cell Fitness

Possessing SRC appears to be essential for T cell stemness. However, what is spare respiratory capacity? Originally defined in terms of neuronal dysfunction, SRC highlights a cell’s potential to produce ATP beyond basal needs [16,52]. Neuronal dysfunction and potential neurodegeneration occur when the SRC is lower than expected and neurons are unable to address stress stimuli with increased ATP production [52,53,54,55,56,57,58,59]. Interestingly, efforts to replenish mitochondria often increase the SRC and may have significant ramifications for slowing the debilitating effects of neurodegeneration [24,53,56,57]. Consequently, one potential source of SRC is simply mitochondrial mass. In the case of CAR T, Kawalekar finds a greater number of mitochondria in the stem-like CD-19 BBζ constructs, with high SRC when compared to the effector like CD-19 28ζ CAR T cells with much lower SRC [19]. Mitochondrial size and fusion may also contribute to SRC. In the gain of function screen from Ye et al. mentioned earlier, *PRODH2* expression enhances SRC while reducing glycolysis in CD22 CAR T cells. This links with increased mitochondrial mass as well as several alterations in metabolic pathways, most notably proline and arginine metabolism [45]. When Tumor Infiltrating Lymphocytes (TILs) are treated with checkpoint inhibitors and co-stimulated with the TNFR binding protein 4-1BB, these T cells raise the SRC, exhibit both mitogenesis and mitochondrial fusion, and provide greater anti-tumor activity than TILs treated with checkpoint inhibitors alone [60]. Memory T cells not only have greater SRC than Effector T cells but contain larger mitochondria too [15,61]. In fact, in Adoptive Cellular Therapy (ACT) models, maintaining mitochondrial fusion in T cells by treating them with the mitochondrial division 1 (DRP1) inhibitor mdivi-1 (mdivi) leads to reduced tumor volume. This group went on to show another feature of SRC in these cells; it is driven by oxidation of fat [61]. Spare respiratory capacity has been linked to the fuels funneling to the mitochondria. Fat oxidation may be paramount to producing memory T cells [15,62]. In addition, amino acids such as glutamine may contribute to mitochondrial fitness and the strength of the immune response to cancer [47,63]. Mitochondrial polarization state and proton motive force may also play a role in SRC and are likely a function of all these factors [16,17].

## 7. Current Approaches to Advancing T Cell Anti-Cancer Therapies

Presently there are five CAR T FDA-approved cell therapies and four target lymphomas [14] (fda.gov). Efforts are underway to produce CAR T cells that are more robust as well as effective against solid tumors. One exciting new path involves combining the recent successes of bispecific antibodies with CAR T technology [64,65,66]. With this approach, the single-chain variable fragment component of the CAR is disposed of in place of arming the CAR T cell with bispecific antibodies capable of recognizing multiple tumor antigens, hence a “headless” CAR T. The approach produces a T cell with greater tumor specificity, enhanced tumor killing, and reduced patient side effects when compared to previous CAR T treatments. Because intracellular signaling remains under the influence of 4-1BB in this design, the T cells remain bioenergetically fit and exhibit considerable mitochondrial spare respiratory capacity [64].

There are at least 700 new and ongoing trials exploring new forms of CAR T cells (clinicaltrails.gov). With our growing knowledge of cytokine signaling, expansion procedures, bioenergetics, gene manipulation, and other techniques, it can be expected there will be further gains. The ability to tailor T cells to the environment or a uniquely expressed tumor antigen is a recent asset made possible by technologies such as CRISPR. Metabolic profiling can provide great insights into the potential for the next generation of CAR T development by providing insight into the metabolic poise produced by the construct. One major hurdle is tumor evasion, something more apparent with ACT strategies targeting solid tumors. There may not be a “one-size-fits all” CAR T for solid tumors. The tumor microenvironment often initiates immune cell dysfunction and apoptosis, so one strategy to encourage a robust immune response to solid tumors is via immune checkpoint inhibition [67]. Interestingly, this approach also influences bioenergetics. In a progressive sarcoma-like tumor microenvironment, for example, the tumor out competes the invading T cells for glucose [37,68]. Many tumors have a high reliance on glycolysis, and therefore require ample glucose. With α-PD1 treatment blocking the programmed cell death protein, not only are T cells more capable of preventing progressive tumor growth, but they also increase rates of glucose uptake and increase both mitochondrial and glycolytic production of ATP [37]. However, α-PD1 alone may not be enough. Menk et al. explored combining CD-137, also known as 4-1BB, with α-PD1 as a potential means of further empowering tumor infiltrating lymphocytes to defeat cancer [60]. The protein 4-1BB is a tumor necrosis factor receptor expressed on activated CD4+ T cells [69]. When applied to CD8+ T cells, 4-1BB increases the SRC of these cells compared to controls and CD28 stimulation. The increased SRC results from p38-MAPK and PGC1a activation, both pathways involved in mitogenesis. Thus, 4-1BB provides the infiltrating T cells with the mitochondria needed to battle cancer. In combination, αPD1 and 4-1BB stimulated CD8+ T cells become superior anti-cancer agents when compared with single treatments in animal models of B16 melanoma, a model for solid tumors [60]. It should be noted that p38 is an attractive target. In a recent screen, p38 has been identified as a key component of T cell activation. In this case, pharmacological inhibition of p38 signaling benefits T cell Physiology for ACT, resulting in increased SRC, increased glucose uptake, stemness, and persistence. Further, in ACT animal models of solid tumor, researchers observed increased survival rates among animals infused with T cells expanded under p38 inhibition [70]. Clearly, more work needs to be done in understanding these pathways. Assessing mitochondrial function once again appears invaluable to assessing the T cell potential. Metabolism is the common thread.

Historically, manipulating metabolism has not been the primary target for most immuno-oncologists. Previous research unequivocally demonstrates a link between metabolic phenotype and immune cell fate [15,61,71,72,73], and evidence continues to arise linking T cell metabolic poise to significant anti-tumor behavior [14,74]. In fact, therapies designed to influence T cell fate, such as α-PD1 and α-CTLA-4, significantly impact bioenergetics, shifting T cells to mitochondrial dependence, albeit through different mechanisms [44,68,75,76]. Altogether, these findings provide a framework for current and future immuno-oncologists to design strategies incorporating metabolic manipulation into the design of CAR T cells. For example, ex-vivo inhibition of glycolysis potentiates CD8+ T cell antitumor behavior [74]. Most approaches to producing effective CAR T cells eventually route through mitochondria, highlighting the importance of mitochondrial mass, mitochondrial function, redox potential, ROS, and the role mitochondria may play in other anti-cancer signaling cascades. Using strategies that produce and stabilize mitochondria such as TFAM- or PGC-1α-driven mitogenesis, as well as nutrient-conditioned expansion, may help further adoptive cellular therapy.

In addition to CAR T technology, current genetic engineering techniques also make possible the production of other chimeras such as CAR D, CAR M, and CAR NK, and even δγ T cells in the fight against cancer. In fact, the rare γδ T cells have shown promise for effectively and safely infiltrating solid tumors with much less if any inflammation [77,78]. Because of their rarity, little is yet known about the metabolism of these cells, although they require glutamine metabolism for cytokine production and activation, notably Il-17 [79]. Early indications suggest that simple dendritic cell modification may be capable of producing highly sensitized T cells. Treating dendritic cells with the CD137 ligand (4-1BB) enhances the inflammatory response of neighboring T cells, enhances T cell bioenergetics, and results in greater lysis of target infected cells; in the cases of hepatitis and Epstein–Barr-infected HepG2 cells, viruses associated with cancer [80]. While not directly linked to genetic engineering, the important take home is the ability to modulate an innate immune cell to tune T cells, another weapon against cancer. Natural killer cells, another innate immune cell, are currently among the most recent immune cells under investigation in the fight against cancer. Unlike T cells, natural killer cells mount a defense against pathogens without previous exposure. They act quickly, are motile, can potentially invade solid tumors, and can easily be produced in the dish. These cells exhibit antigenicity against invading tumors as well. So why are these cells not inherently defeating cancer? What needs to be changed? Tumor-associated natural killer cells, or TANKs, suffer an inability to remain active in the tumor microenvironment. Poznanski et al. demonstrated impaired killing efficiency in TANKs when compared with natural killer cells isolated from peripheral blood of human subjects. TANKS suffered bioenergetically, too, with less glycolysis and less SRC when compared to the peripheral blood natural killer cells [81]. Infiltrating natural killer cells suffer from shortened mitochondria [82], and impaired mitochondria may explain the reduced SRC and poor functionality. Expanding the TANKs in the presence of IL-21 to stimulate STAT3 improves the bioenergetic profile to that seen in the peripheral blood natural killer cells and leads to greater killing efficiency against solid tumors, further highlighting the important role of bioenergetics in immunotherapeutic engineering [81]. Of note, the success in restoring killing ability in TANKs via STAT3 signaling appears to rely on metabolic flexibility. IL-21-stimulated natural killer cells were not as reliant on glucose for fuel as their control counterparts [81]. Of note, treating natural killer cells in culture with mdivi-1 also restores metabolic potential and improves hepatoma (solid) tumor killing [82]. A drawback of natural killer cell anti-tumor activity is a lack of persistence. These are short lived cells. Much like with CAR T engineering, identifying, and targeting intracellular signaling pathways can be used to tune natural killer and other cells to a desired outcome. Natural killer cells carrying a deletion in cytokine inducible factor 2 are capable of long-term growth beyond that of a control natural killer cell in the presence of IL-15, up to at least three weeks. With cytokine inducible factor 2 deleted, the natural killer cell response to IL-15 promotes expansion without contraction, longevity, and effective acute myeloid tumor killing. The expanded cells also show increased levels of glycolysis, mitochondrial activity, and increased SRC when compared to unstimulated and wildtype natural killer cells weeks after expansion. They are metabolically fit, and they persist [83]. In the future, it is not unlikely that combinations of immune cells and immune-active agents will be employed, tailored to the specific cancer. The technology is presently revisiting Coley’s work but skipping the infection with disease and going right to activated source cells. This is why Coley is considered the “Grandfather of Immunotherapy”.

## 8. How Safe Is CAR T?

Coley’s approach stimulates the immune system with a disease to “trick” the immune system into fighting off a tumor. A sickness is introduced to fight a cancer. The safety of the approach becomes an issue. Modern approaches with CAR T cells are more directed toward the tumor to prevent harm to normal cells, but the immune system is a complex, highly evolved system involving numerous cell types, signaling cascades, and dynamic functional changes throughout the infection. Current third, fourth, and now fifth generation CAR T attempt to target antigens expressed solely by the cancer cells. On- and off-target effects of CAR T are always a concern. On-target effects refer to the CAR T attacking a healthy cell also expressing the target antigen, for example, B cells express CD19. Off-target effects involve CAR T cells damaging healthy cells that do not express the target antigen. In both cases, the hope is that the CAR T is more damaging to the tumor than to healthy tissue. For the patient, several adverse effects may present such as fever, hypoxia, hypotension, tachycardia, etc., many of these resulting from cytokine release syndrome, commonly referred to as cytokine storm [84,85,86]. It is not fully clear how cytokine release syndrome is initiated, although there is evidence that engineered CAR T cells may stimulate neighboring cells to induce cytokine release and signaling [87]. Highly specific, targeted CAR T cell activation, such as with bi-specific antibodies, is one approach to minimize activation of other immune cells [38,64,65,88]. Ample research is dedicated to examining cytokine release syndrome because it is not only an issue with CAR T therapy, but with many infectious diseases. Activation of innate immune cells results in expression of Il-1b, Il-6, and other proinflammatory cytokines. This is often associated with increases in glycolytic activity, thereby not only posing a threat to healthy tissue, but also competing with the CAR T cells for resources. Managing innate immune cell bioenergetics is one approach to limiting this hyperinflammation [89]. Targeting the cytokines involved in inflammatory responses is another approach [90]. In both of these cases, reduced inflammation is associated with decreased mitochondrial respiration within the innate cells.

## 9. Stay Tuned

The future of defeating cancer with a person’s own immune cells continues to hold great promise. The first long-term evaluation of CAR T treatment for individuals with leukemia is an overwhelming success [48], success that depended on a numerous of factors, notwithstanding metabolic fitness and a good understanding of bioenergetics. The data and information provided by these studies on CAR T cells provide a roadmap for the study, profiling, and prediction of successful future ACT treatments, thereby increasing the speed to develop new remedies. Immune cell metabolic fitness is a key component to this success.

## Data Availability

No new data were created or analyzed in this study. Data sharing is not applicable to this article.
